# Psychometric properties of the Brazilian version of the Child Perceptions Questionnaire (CPQ_11–14_) – short forms

**DOI:** 10.1186/1477-7525-7-43

**Published:** 2009-05-17

**Authors:** Cíntia S Torres, Saul M Paiva, Miriam P Vale, Isabela A Pordeus, Maria L Ramos-Jorge, Ana C Oliveira, Paul J Allison

**Affiliations:** 1 Department of Pediatric Dentistry and Orthodontics, Faculty of Dentistry, Federal University of Minas Gerais, Av. Antônio Carlos 6627, Belo Horizonte, MG, 31270-901, Brazil; 2 Division of Public Health and Society, Faculty of Dentistry, McGill University, 3640 University Street, Montreal, QC, H3A 2B2, Canada; 3 Pediatric Dentistry and Community Health Department, Faculty of Dentistry, University of Valley of Jequitinhonha and Mucuri, Campus II, Rodovia MGT 367, Km 583, 5000, Diamantina, MG, 39100-000, Brazil

## Abstract

**Background:**

The need to evaluate the impact of oral health has led to the development of instruments for measuring oral health-related quality of life (OHQoL). One such instrument is the Child Perceptions Questionnaire (CPQ_11–14_), developed specifically for 11-to-14-year-old children. As this questionnaire was considered long (37 items), shorter forms were developed with 8 (Impact Short Form: 8 – ISF:8) and 16 items (Impact Short Form: 16 – ISF:16) to facilitate use in the clinical setting and population-based health surveys. The aim of the present study was to translate and cross-culturally adapt these CPQ_11–14 _short forms for Brazilian Portuguese and evaluate the measurement properties of these versions for use on Brazilian children.

**Methods:**

Following translation and cross-cultural adaptation, the ISF:8 and ISF:16 were tested on 136 children from 11 to 14 years of age in the city of Belo Horizonte, Brazil. The instrument was administered by a trained researcher who also performed clinical examinations. The measurement properties (i.e. criterion validity, construct validity, internal consistency reliability, test-retest reliability) were determined. Discriminant validity was tested between groups, which were divided into children with no cavities and no malocclusion; children with cavities and without malocclusion; and children with malocclusion and without cavities.

**Results:**

The mean total score was 6.8 [standard deviation (SD) 4.2] for the ISF:8 and 11.9 (SD 7.6) for the ISF:16 (p < 0.001). Statistically significant associations were found between oral abnormalities and the subscales of the ISF:8 and ISF:16 (*p *< 0.05). Both test-retest stability and internal consistency, as measured by the intra-class correlation coefficient (ICC) (ISF:8 = 0.98 and ISF:16 = 0.97) and Cronbach's alpha (ISF:8 = 0.70 and ISF:16 = 0.84) proved to be adequate. Construct validity was confirmed from the correlation between the short form scores and oral health and overall well-being ratings. The score on the short forms of the CPQ_11–14 _was able to discriminate between different oral conditions. Criterion validity was satisfactory (p < 0.05).

**Conclusion:**

The Brazilian versions of CPQ_11–14 _ISF:8 and ISF:16 have satisfactory psychometric properties, similar to those of the original instrument.

## Background

Little more than twenty years ago, there were no methods for assessing the impact of oral-facial problems on the daily living of individuals. The need to determine the repercussions of oral abnormalities has led to the development of instruments for measuring oral health-related quality of life, which have been used with increasing frequency in dental studies [1]. When associated to clinical data, oral health-related quality of life measures provide important information for improvements in the planning and direction of health actions. Self-perception regarding oral health status can be addressed in such a way as to encourage individuals to adopt healthy behavior [2].

A number of questionnaires for assessing the correlation between oral health and quality of life have been developed and are being cross-culturally adapted and administered in studies carried out in different countries. However, most are directed toward the adult population [1-6]. The first specific instruments for children were developed by Jokovic et al. [7,8]. These authors developed the Child Oral Health Quality of Life (COHQoL), a set of questionnaires that aim to measure the impact of oral health abnormalities on the quality of life of children between six and 14 years of age (Child Perceptions Questionnaire – CPQ) as well as their families (Family Impact Scale – FIS) and the perception of parents/caregivers regarding the oral health of their children (Parental-Caregiver Perceptions Questionnaire – P-CPQ). These instruments encompass the following subscales: oral symptoms, functional limitations, emotional wellbeing and social wellbeing. They also includes sub-subscales addressing school interaction and recreation activities. These questionnaires were developed and validated in Canada in the English language and their psychometric properties were deemed satisfactory, indicating their validity [7,8].

Cross-cultural adaptation is necessary in order to make viable the collection of information in other cultures. The CPQ_11–14 _has been tested and validated on children in New Zealand, England, Saudi Arabia, Brazil e China [9-15]. The original measure is made up of 37 items, but is considered long and difficult to administer in clinical settings and population-based studies [12-14]. In order to facility the applicability of the measure, Jokovic et al. [16] developed short versions of the CPQ_11–14 _for children in this age group, giving rise to the Impact Short Forms ISF:16 and ISF:8. The authors have determined the psychometric properties of these short forms to be satisfactory, but state that these measures must be validated and employed in other cultures, involving clinical and population-based samples of children and adolescents in different countries [17].

The aim of the current study was to translate and cross-culturally adapt to Brazilian Portuguese the ISF:8 and ISF:16 measures as well as assess the reliability and validity of these versions for use on Brazilian children between 11 and 14 years of age.

## Methods

### Short forms of the Child Perceptions Questionnaire – ISF:8 and ISF:16

The ISF:8 and ISF:16 questionnaires are short forms of the CPQ_11–14_developed in Canada by Jokovic et al.[16]. These short forms were developed from the inclusion of the items on the full lenght version that obtained the highest scores, indicating a greater impact on the quality of life of children. The items address the frequency of events in the previous three months. The measures are structurally composed of 8 and 16 items distributed among 4 subscales: oral symptoms, functional limitations, emotional wellbeing and social wellbeing. A 5-point Likert scale is used, with the following options: 'Never' = 0; 'Once/twice' = 1; 'Sometimes' = 2; 'Often' = 3; and 'Every day/almost every day' = 4.

The authors also included two questions asking the children for a global rating of their oral health and the extent to which their oral health affects their overall well-being [7]. These questions are: 'Would you say that the health of your teeth, lips, jaws and mouth is...?' and 'How much does the condition of your teeth, lips, jaws or mouth affect your life overall?' These global ratings had a five-point response format. The responses were scored as follows: for global rating of oral health, (0) excellent, (1) very good, (2) good, (3) fair and (4) poor; and for overall well-being, (0) not at all, (1) very little, (2) somewhat, (3) a lot and (4) very much.

The short forms of the CPQ_11–14 _scores are computed by summing the item scores. Separate scores for each of the four subscales can also be computed. As there are 16 and 8 questions, the final scores range from 0 to 64 and 0 to 32, for which a higher score denotes a greater degree of the impact of oral conditions on the quality of life.

### Adaptation and translation of the CPQ_11–14 _short forms

In order to measure the OHRQoL of children in Brazil, the questionnaires were subjected to translation and cross-cultural adaptation to Brazilian culture [18,19]. Based on standard recommendations, two bilingual translators with experience in translating health-related questionnaires (a Brazilian fluent in the English language and a native English speaker fluent in Portuguese) carried out two independent translations. To determine concept and item equivalence, the translated versions were analyzed by a group of specialists, who drafted synthesized versions. Attention was given to the meaning of the words in the different languages in order to obtain similar effects on respondents from different cultures, seeking to identify possible difficulties in understanding the questionnaires. These versions were then backtranslated by a bilingual translator whose native language was English and who had no access to the original versions. To assess the equivalence between the original and backtranslated questionnaires, a Brazilian translator whose native language was Portuguese and who was fluent in English carried out a third assessment between the original and backtranslated versions. Operational equivalence was determined on a sample of 37 children between 11 and 14 years of age who did not make up part of the main sample. The Brazilian versions of CPQ_11–14 _short forms achieved satisfactory concept and semantic equivalence when compared to the original instruments, proving the questionnaires could be applied for the assessment of reliability and validity of these versions on Brazilian children.

### Assessment of validity and reliability of the Brazilian version of the Impact Short Forms derived from the CPQ_11–14_

The study was conducted in Belo Horizonte, capital city of the state of Minas Gerais, Brazil. Data collection was carried out through the administration of the ISF:8 and ISF:16 measures in the self-applicable format to 136 male and female public school children between 11 and 14 years of age. Participants completed both the ISF:8 and ISF:16 separately. Parents/guardians and children read and signed terms of informed consent prior to participation in the study. The study received approval from the Research Ethics Committee of the Federal University of Minas Gerais, Brazil.

Children in dental treatment during the study, those with the presence of dental trauma and those with the simultaneous presence of carious lesion and malocclusion were excluded from the study. The criteria of the World Health Organization (WHO) [20] were used for the assessment of dental caries and the Dental Aesthetic Index (DAI) [21] was used for the assessment of malocclusion.

The standardization process was carried out with 16 children for the evaluation of intra-examiner agreement regarding caries and malocclusion. Minimal and maximal Kappa values for dental caries were 0.91 and 0.94, respectively. The intra-class correlation coefficient was used for agreement on the diagnosis of malocclusion, achieving a value of 0.84.

For discriminant validity, the children were divided into three groups according to the data from the oral examination: Group 1 – children with no cavities and no malocclusion; Group 2 – children with cavities and without malocclusion; and Group 3 – children with malocclusion and without cavities.

After the oral examination, the 136 participants completed the first questionnaire (ISF:8) and following a 45-day interval, the same children completed the second questionnaire (ISF:16). The data were grouped in a databank and the SPSS software program (version 15.0. SPSS Inc., Chicago, IL, USA) was used for statistical analysis. Descriptive analyses were performed (mean, standard deviation, analysis of total and individual ISF:8 and ISF:16 subscale scores) in order to generate total and subscale scores for each participant.

Reliability was assessed by tests of internal consistency and stability. The degree of homogeneity of the scale was assessed using Cronbach's α coefficient to determine the extent of agreement between all possible subsets of questions [22]. Item/total score and inter-item score correlations were also determined.

Stability was evaluated using the test-retest approach. The intra-class correlation coefficient (ICC), with a 95% confidence interval, was calculated based on the repeated interview of a sub-sample of 86 participants chosen among those 136 that made up the main sample, using the following criteria: ≤ 0.40 (weak), 0.41–0.60 (moderate), 0.61–0.80 (good), 0.80–1.00 (excellent) [23].

Construct validity was analyzed through convergent validity and discriminant validity. Spearman's correlation coefficient was used to test convergent validity. Associations were analyzed between total scores and subscales scores with the oral health and well-being global indicators for both the ISF:8 and ISF:16.

Discriminant validity was tested by comparing the mean total scores on the questionnaire and subscales between the groups. As the ISF:8 and ISF:16 scores were not normally distributed, the nonparametric Kruskal-Wallis test was used to evaluate the difference in mean scores between the three groups. The level of significance was set at 0.05.

Criterion validity was obtained in order to determine whether the instruments measure the same construct. For such, the total score and subscale scores were correlated between the ISF:8 and ISF:16 questionnaires using Spearman's correlation coefficient.

## Results

Among the 154 children initially selected, 136 individuals participated in the study. The remaining children were excluded for undergoing dental treatment during the study (n = 4), presenting dental trauma on the examination day (n = 2) and having cavities and malocclusion simultaneously (n = 12). The final sample included 56 boys (41.2%) and 80 girls (58.8%), totaling 136 individuals. Mean age was 12.7 years (SD = 1.1), distributed in the following manner: 25 children were 11 years old (18.4%), 32 were 12 years old (23.5%), 30 were 13 years old (22.1%) and 49 were 14 years old (36.0%). The children were divided into Group 1, 56 (41.2%) children with no cavities or malocclusion; Group 2, 34 (25.0%) children with cavities and without malocclusion; and Group 3, 46 (33.8%) children with malocclusion and without cavities.

The total ISF:8 score ranged from 0 to 18, with a mean score of 6.8 (SD = 4.2). The total ISF:16 score ranged from 0 to 38, with a mean score of 11.9 (SD = 7.6). On both questionnaires, the frequency of a total score of zero was 2.9%. No child achieved the maximal possible score on either questionnaire (Table 1).

**Table 1:** Descriptive statistics for the CPQ_11–14 _short forms ISF:8 and ISF:16 (n = 136)

Short-Forms:	Range of possible Scores	Mean (SD)	Range of obtained scores	% with score of 0	% with max score
ISF:8	0 – 32	6.8 (4.2)	0 – 18	2.9	0.0
ISF:16	0 – 64	11.9 (7.6)	0 – 38	2.9	0.0

### Reliability

Analysis of Cronbach's alpha coefficient revealed values near or above 0.70 for total scores, indicating satisfactory internal consistence. Subscales scores were distributed in a heterogeneous manner on both the ISF:8 and ISF:16. Reproducibility and stability of the measures were confirmed by the ICC, demonstrating excellent correlations for the total and subscale scores on both questionnaires (Table 2).

**Table 2:** Reliability statistics for total scale and subscales: Short Forms of the CPQ_11–14 _ISF:8 and ISF:16

Variable	Number of items	Cronbach's alpha (n** = **136)	Intraclass correlation coefficient (95% CI)* (n **= **86)
Total scale			
ISF:8	8	0.70	0.98 (0.97–0.99)
ISF:16	16	0.84	0.97 (0.94–0.97)
Subscale			
Oral Symptoms			
ISF:8	2	0.40	0.98 (0.96–0.98)
ISF:16	4	0.63	0.93 (0.89–0.95)
Functional limitations			
ISF:8	2	0.41	0.96 (0.95–0.98)
ISF:16	4	0.50	0.94 (0.91–0.96)
Emotional well-being			
ISF:8	2	0.71	0.99 (0.98–0.99)
ISF:16	4	0.70	0.96 (0.93–0.97)
Social well-being			
ISF:8	2	0.32	0.99 (0.93–0.99)
ISF:16	4	0.68	0.95 (0.92–0.96)

* Two-way random effect model: *p *< 0.001 for all values

### Construct validity

The ISF:8 and ISF:16 had statistically significant, positive correlations between total and subscale scores and the global indicators oral health and well-being, with a better correlation to the oral healthrating. The correlation between the global indicators and ISF:8 subscales was not statistically significant between the functional limitations subscale and the well-being global indicator. The remaining subscales, however, were positively correlated to the global indicators (Table 3).

**Table 3:** Construct validity: rank correlations between total scale and subscale scores, and global rating of oral health and overall wellbeing on ISF:8 and ISF:16 (n = 136)

	**Global rating**			
	
	Oral health		Overall wellbeing	
	
	r *	p-value	r *	p-value
Total scale				
ISF:8	0.47	<0.001	0.32	<0.001
ISF:16	0.49	<0.001	0.33	<0.001
Subscale		<0.001		
Oral Symptoms		<0.001		
ISF:8	0.35	<0.001	0.37	<0.001
ISF:16	0.53	<0.001	0.27	<0.001
Functional limitations		<0.001		
ISF:8	0.31	<0.001	0.09	0.26
ISF:16	0.35	<0.001	0.20	0.02
Emotional well-being		<0.001		
ISF:8	0.35	<0.001	0.31	<0.001
ISF:16	0.40	<0.001	0.34	<0.001
Social well-being		<0.001		<0.001
ISF:8	0.35	<0.001	0.17	<0.001
ISF:16	0.28	<0.001	0.29	<0.001

* Spearman's correlation coefficient

### Discriminant validity

Discriminant validity was determined by comparing scores between the clinical groups. Mean total scores were higher among the groups with oral abnormalities than the groups without abnormalities, revealing that the instruments were capable of clinically discriminating between the different groups. Statistically significant results were obtained between the subscales of the instruments and the groups studied, except the functional limitations subscale on the ISF:8 and the emotional well-being subscales on both the ISF:8 and ISF:16 (Table 4).

**Table 4:** Discriminant validity of the ISF:8 and ISF:16: overall and subscale scores for children with no cavities or malocclusion (Group 1); with cavities and without malocclusion (Group 2); and with malocclusion and without cavities (Group 3) (n = 136)

	**Group 1 (n = 56)**		**Group 2 (n = 34)**		**Group 3 (n = 46)**		
		
	mean ± SD	median	mean ± SD	median	mean ± SD	median	p-value*
Total scale							
ISF:8	5.66 ± 3.73	5.00	8.50 ± 4.61	8.00	6.93 ± 4.23	6.00	<0.001
ISF:16	9.63 ± 7.78	7.00	12.94 ± 5.55	13.00	13.98 ± 8.4	12.00	<0.001
Subscale							
Oral Symptoms							
ISF:8	2.07 ± 1.33	2.00	3.09 ± 1.24	3.00	2.15 ± 1.60	2.00	<0.001
ISF:16	3.43 ± 2.45	3.00	5.18 ± 2.02	5.50	4.28 ± 2.50	4.00	<0.001
Functional limitations							
ISF:8	1.80 ± 1.86	1.00	2.59 ± 1.87	2.50	1.74 ± 1.58	2.00	0.06
ISF:16	2.70 ± 2.52	2.00	3.68 ± 1.99	3.00	3.78 ± 2.40	4.00	0.01
Emotional well-being							
ISF:8	1.02 ± 1.43	0.00	1.47 ± 1.46	1.00	1.13 ± 1.37	1.00	0.22
ISF:16	2.16 ± 2,36	2.00	2.68 ± 2.40	2.00	3.15 ± 2.54	2.50	0.07
Social well-being							
ISF:8	0.77 ± 0.99	0.00	1.35 ± 1.39	1.00	1.91 ± 1.50	2.00	<0.001
ISF:16	1.34 ± 2.20	0.00	1.41 ± 1.37	1.00	2.76 ± 2.56	2.00	<0.001

*p-values obtained from Kruskal-Wallis test

### Criterion validity

The criterion validity was obtained through the correlation of the questionnaires to one another, revealing statistically significant, positive correlations between the total and subscale scores of the two measures (Table 5).

**Table 5:** Criterion validity: rank correlations between scores of the total scale and subscales on the ISF:8 and ISF:16 (n = 136)

	r*	p-value
Total scale	0.47	<0.001
Subscales		
Oral Symptoms	0.44	<0.001
Functional limitations	0.45	<0.001
Emotional well-being	0.25	<0.001
Social well-being	0.37	<0.001

* Spearman's correlation coefficient

## Discussion

The ISF:8 and ISF:16 questionnaires were selected for translation and cross-cultural adaptation to the Portuguese language as well as the assessment of reliability and validity for administration to children in Brazil. A number of studies consider that measures derived from the impact method are more appropriate that those derived from mathematical regression due to the fact that the former method selects items of greater importance – those that identify a greater impact on individuals [24-26].

The full length version of the CPQ_11–14 _cross-culturally adapted to Brazilian Portuguese proved valid and reliable for its use on Brazilian children [15]. It was therefore believed that the short forms would provide greater applicability of the measure in clinical and population-based studies through the reduction in time and cost during data collection as well as a reduced risk of losses [16].

The ISF:8 and ISF:16, measures translated and cross-culturally adapted to the Portuguese language, demonstrated satisfactory internal consistency and test-retest reliability. Cronbach's alpha coefficient for the total scores revealed an adequate homogeneity of the items on the two measures (0.70 and 0.84). This finding is similar to that described during the development and validity of the original short forms (0.71 and 0.83) [16] and that validated in New Zealand (0.73 to 0.86) [27], whereas the original study on the full length version of the CPQ_11–14 _achieved a value of 0.91 [7]. In subsequent validations of the full length version, the results were 0.81 in Saudi Arabia [11], 0.86 in Brazil [15] and 0.89 in China [12].

Cronbach's alpha coefficient ranged from 0.32 to 0.71 for the ISF:8 subscales and from 0.50 to 0.70 for the ISF:16 subscales. These results are heterogeneous, but higher than those obtained by Jokovic et al. [16] (0.31 to 0.47 for ISF:8 and 0.30 to 0.57 for ISF:16). The authors state that the heterogeneous values of internal consistency among the subscales may be related to the small number of items that make up the questionnaires. A small number of items on a questionnaire can also affect its content validity. Even when relevance remains intact, the construct validity may be compromised due to the omission of individual problems [16,26]. In the present study, the short forms achieved acceptable construct validity, demonstrating a positive correlation between the global indicators and total score on the ISF:8 and ISF:16. Jokovic et al. [16] found correlations of 0.19 and 0.39 between total score and the global indicators for the ISF:8 and correlations of 0.21 and 0.40 for the ISF:16, which are similar to the findings of the present study. However, the measures were better correlated with the oral health rating in the present study than the well-being rating. This is the opposite from what occurred in the original study on the short forms [16] and the Brazilian study on the long form [15], whereas this finding is similar to that described in the long form validation study carried out in Saudi Arabia [11] and in New Zealand [27].

Statistically significant associations were found between the ISF:8 subscales and the oral health and general well-being ratings. However, the association between the functional limitations subscale and the well-being rating were not statistically significant. A large portion of the children, even those without cavities, reported difficulty in eating/drinking hot or cold foods and beverages. As the study was carried out at a school, it was not possible to detect conditions that could only be visualized radiographically. All associations between the ISF:16 subscales and the global indicators oral health and well-being were statistically significant. Further studies using qualitative approach are necessary to investigate, in depth, the meaning of the items of the CPQ.

To confirm discriminant validity, the mean total ISF:8 and ISF:16 scores were determined. The results were similar to those described by Jokovic et al. [16] and by Page et al. [27], demonstrating that children with oral health abnormalities achieved higher mean total scores on each questionnaire, which signifies the greater impact of these conditions on the quality of life of these individuals. Inverted results were found between the Brazilian versions of the ISF:8 and ISF:16 in the comparison of the groups with carious lesions (Group 2), malocclusion (Group 3) and the group without these conditions (Group 1). On the ISF:8, the mean total score for Group 2 was greater than that of Group 3, whereas the opposite occurred with ISF:16. This finding is likely due to the small number of items on ISF:8. Regarding the analysis of the subscales taken separately, no statistically significant association was found between the groups and the functional limitations and emotional well-being subscales on either the ISF:8 or ISF:16. The remaining subscales had the same tendency as the total score, achieving significantly higher mean values in the groups with oral abnormalities.

As the short versions of the CPQ_11–14 _were only developed recently, the comparison of the results obtained in the present study is hindered by the lack of studies that have validated and administered the ISF:8 and ISF:16. Therefore, the results were compared to the data from the cross-cultural validations of the long form and the cross-cultural validation of the New Zealanders' short forms.

The criterion validity revealed a statistically significant, positive association between total score (0.47) and subscale scores on the two measures: 0.25 to 0.44 (p < 0.001), suggesting that the instruments measure the same construct (Table 5). In the study by Jokovic et al. [16], the short forms were strongly correlated with the long form, with results ranging from 0.87 to 0.96 (p < 0.001), indicating that the short forms can be used to substitute the full length form of the CPQ_11–14_. Although the Brazilian version of both short forms (ISF:8 and ISF:16) exhibited satisfactory psychometric properties, the ISF:16 had a better performance than the ISF:8, which is likely due to the small number of items on the ISF:8.

The validation of the short forms of questionnaires is important, as it facilitates their use in population-based surveys with large sample size. The results of the present study provide evidence of the satisfactory properties of reliability, construct validity and discriminant validity of the Brazilian version of the short forms of the Child Perceptions Questionnaire for children between 11 and 14 years of age, thereby demonstrating their applicability in this population.

## Conclusion

The Brazilian versions of the short forms of the CPQ_11–14 _(ISF:8 and ISF:16) demonstrated acceptable reliability and validity, thereby confirming the applicability of these measures on Brazilian children between 11 and 14 years of age. The psychometric properties were found to be satisfactory. However, further research is necessary for the confirmation of these properties in other populations and settings.

## Abbreviations

OHRQoL: Oral Health-Related Quality of Life; CPQ: Child Perceptions Questionnaire; ISF: Impact Short Form; ICC: Intra-class Correlation Coefficient; COHQoL: Child Oral Health Quality of Life Questionnaire; FIS: Family Impact Scale; P-CPQ: Parental-Caregiver Perceptions Questionnaire; WHO: World Health Organization; DAI: Dental Aesthetic Index; SPSS: Statistical Package for Social Sciences.

## Competing interests

The authors declare that they have no competing interests.

## Authors' contributions

CT, SP, MP, IP and PA conceptualized the rationale and designed the study. CT, MRJ, AO performed the data collection, statistical analysis and interpretation of the data. CT, SP, MRJ and PA conducted the literature review and drafted the manuscript. All authors read and approved the final manuscript.
